# Emerging Applications of Liquid Biopsies in Ovarian Cancer

**DOI:** 10.7759/cureus.49880

**Published:** 2023-12-03

**Authors:** Urvi S Chauhan, Mangesh G Kohale, Neha Jaiswal, Rashmi Wankhade

**Affiliations:** 1 Pathology, Datta Meghe Medical College, Datta Meghe Institute of Higher Education & Research (Deemed to be University), Wardha, IND

**Keywords:** non-invasiveness, circrna, lncrna, mirna, ovarian cancer, tumor-educated platelet, circulating exosomes, circulating tumor cells (ctcs)

## Abstract

Liquid biopsy is a new diagnostic tool in precision oncology that can be used as a complementary or alternative method to surgical biopsies. It is a cutting-edge sampling technique that examines distinct cancer components, such as exosomes and circulating tumor cells discharged into the peripheral circulation, to identify tumor biomarkers through various methods, including polymerase chain reaction (PCR). Liquid biopsy has several benefits, including its non-invasiveness and practicality, which permit serial sampling and long-term monitoring of dynamic tumor changes. Ovarian cancer (OC), the most lethal gynecologic malignancy in the world, is typically diagnosed at stages II and III, which makes recovery and treatment extremely difficult. Relapsed OC and chemotherapy resistance of ovarian tumor cells are other clinical challenges. Although liquid biopsy is not a routinely used diagnostic test, it should be utilized in the diagnosis and prognosis of OC for early detection and treatment. It is less intrusive than conventional tissue biopsies, allowing for the continuous collection of serial blood samples to track cancer development in real time. Before therapeutic application, more investigation is required to pinpoint the particular release processes, the source tissue, and the biological significance of the bulk of liquid biopsy contents.

## Introduction and background

Ovarian cancer (OC) ranks as the seventh most prevalent gynecological cancer, with almost 300,000 new cases per year [1]. Northern and Central/Eastern Europe has the most significant incidence rate of OC, according to the International Agency for Research on Cancer (IARC). OC may not show signs in the early stages. Therefore, 70% of cases are discovered when they are more advanced and have metastases [2]. The overall survival rate is about 30% [3]. However, OC in its early stages has a good prognosis, with five-year survival rates as high as 95%. High-grade serous ovarian cancer (HGSOC), a type II tumor, is aggressive and has high genetic instability [4]. These are linked to high mutation rates in the homologous recombination genes tumor protein 53 (TP53) and the somatic and germline breast cancer gene 1/2 (BRCA1/2). The choice of the best-targeted therapy can be influenced by determining the particular mutational makeup of cancer [5]. The significant rates of morbidity and mortality associated with OC are attributed to the delay in diagnosis and the ineffectiveness of surgical or pharmaceutical treatments [6]. Over the past few decades, efforts have been made to find diagnostic, prognostic, or predictive biomarkers to improve the treatment of OC [7]. Tissue biopsies are impractical because they are invasive and require only exclusive samples with low sensitivity. Due to the limitations of current screening and detection techniques, researchers are still looking for more accurate and sensitive OC biomarkers. A liquid biopsy is a test that detects cancer cells or circulating deoxyribonucelic acid (DNA), including tumor cells. Liquid biopsy has several benefits, including its non-invasiveness and practicality, which permit serial sampling and long-term monitoring of dynamic tumor changes. Liquid biopsy is less intrusive than conventional tissue biopsies, and it allows for the continuous collection of serial blood samples to track cancer development in real time [8]. This review discusses the benefits and present limitations of liquid biopsy in the treatment of OC.

## Review

Components of liquid biopsy

Circulating Tumor Cells

Circulating tumor cells (CTCs) are intricate cancer cells, passively shed from a solid primary or metastatic tumor site and discovered in peripheral blood [9]. According to various biological characteristics, such as positive epithelial markers, negative hematopoietic markers, or physical characteristics, including size, density, deformability, electrical charges, and invasive capacity, several analytical CTC isolation techniques have been created and validated for OC [10,11]. To identify possible micrometastases, pre-neoplastic lesions, tumor heterogeneity, and tumor progression over time in OC, the ability to detect CTCs in the bloodstream is of prognostic significance [11,12]. According to theory, it is difficult to isolate CTCs from peripheral blood samples due to their low concentration [13-15]. CTCs must overcome several challenges to live in the systemic vascular system and propagate to distant organs after being released from the primary tumor [16]. When epithelial cells undergo the epithelial-to-mesenchymal transition (EMT), they lose their polarity, shape, and cell markers, such as the epithelial cell adhesion molecule (EpCAM), and develop the migratory and invasive characteristics of mesenchymal cells [17]. About one million CTCs dissociate daily from a primary model V2 carcinoma site and intravasate into the peripheral circulation by cutaneous invasion. Of these, about 1% are still viable for metastasis [18]. Due to severe environmental hurdles in the bloodstream, including hunger, shear stress, and immunological identification, most CTCs experience apoptosis or necrosis [19]. Through the activation of numerous signaling pathways, such as enhanced growth factor secretion, decreased expression of death receptors, and over-expression of anti-apoptotic ligands, only a tiny percentage of CTCs can survive [18]. The most popular isolation method currently in use and approved by the US Food and Drug Administration (FDA) is the CellSearch detection system. Based on the positive expression of EpCAM, CellSearch uses an immunoaffinity-based isolation approach to find CTC [20]. There was no association between serial CTC enumeration using the CellSearch technology and clinical outcomes in a trial involving patients with newly diagnosed or recurrent OC. More research is needed to improve the identification and separation in OC [15].

Cell-Free and Circulating Tumor DNA

Normal cell-free DNA (cfDNA) is released into the bloodstream by dying or apoptotic cells, whereas circulating tumor DNA (ctDNA) is cfDNA secreted by cancer cells. Following tissue damage caused by strenuous exercise, inflammation, sepsis, surgery, radiation, trauma, or pregnancy, healthy individuals' cfDNA concentrations increase [21-23]. Blood contains more significant amounts of cfDNA than CTCs, making it a good target for liquid biopsy [24]. The unique somatic genetic changes present in tumors help to separate malignant tumor DNA (ctDNA) from non-cancerous tumor DNA (cfDNA) [21,22]. Although a variable percentage of cfDNA (0.01-93%, depending on the tumor size) can come from cancer cells (ctDNA), the majority of cfDNA is anticipated to come from healthy cells [25,26]. Other hypothesized mechanisms of cfDNA release include phagocytosis, neutrophil extracellular trap release (NETosis), active secretion in live cells with nuclear ejection, and repair of excision repair [27-32]. In addition, as a measure of cfDNA integrity, the distribution of DNA fragments of various sizes has implications for disease staging. According to studies, cancer patients had a higher cfDNA integrity than healthy individuals or people with benign diseases [33,34]. Higher levels of necrotic cell death are linked to greater integrity in advanced diseases with larger and faster-growing tumors [33,35]. Organs, such as the liver, spleen, kidney, or lymph nodes, may remove cfDNA [36]. Circulating enzymes in the bloodstream, including factor H, plasma factor VII-activating protease (FSAP), and DNase I, break down cfDNA [37,38]. The researchers revealed that the more sensitive techniques of digital droplet polymerase chain reaction (PCR) and the Safe-Sequencing System (SafeSeqS) increased the percentage of ctDNA detection to up to 80%. Interestingly, according to the study, the five patients with stage I disease had TP53 mutations in lavage samples [39,40]. For ctDNA profiling, other cutting-edge methods have been used, such as peritoneal washing, vaginal sampling, and urine collection. These techniques need to be fully studied to understand their diagnostic value in OC [41].

*Cell-Free Ribonucleic Acid (cfRNA)*​​

A rapid tumor turnover causes high levels of gene transcription and the release of cell-free ribonucleic acid (cfRNA), which is made up of messenger ribonucleic acid (mRNA) and mitochondrial ribonucleic acid (miRNA), into the bloodstream. Plasma, urine, vaginal discharge, and breast milk are the only bodily fluids in which normal and cancer cells release miRNAs [42,43].

Messenger RNA and Mitochondrial RNA 

To prevent degradation and gain excellent blood stability, mRNA and miRNA are packaged in extracellular vesicles, such as exosomes, high-density lipoproteins, platelets, and other ribonucleoprotein complexes [44,45]. Numerous studies have suggested that miRNAs may be involved in apoptosis, metastasis, inhibition of angiogenesis, cell differentiation, and carcinogenesis. MiRNAs may be better suited for the early diagnosis of OC since their synthesis and activation occur more quickly and have longer half-lives than mRNA and proteins [45-48]. According to a study, combined miRNAs can distinguish between OC and other malignancies but not sarcoma or esophageal cancer, such as lung, gastric, breast, liver, colorectal, or pancreatic [49-51].

Circular RNA and Long-Coding RNA

In addition to miRNA, circular RNAs (circRNAs) and long noncoding RNAs (lncRNAs) have shown potential utility as biomarkers for liquid biopsy in OC. CircRNAs and lncRNAs feature covalently closed-loop structures that provide greater stability and resistance to destruction in the peripheral circulation. CircRNAs are numerous and diverse, having a half-life of at least 48 hours, which makes them easier to identify. Although growing evidence links different levels of lncRNA expression (H19, LSINCT5, and ANRIL) with clinical OC progression or response to OC, the diagnostic sensitivity and specificity of lncRNAs have not yet been fully characterized [52-57]. More research is needed to find the most clinically relevant individuals with cancer-rich or specific signatures in OC, as lncRNA has been authorized for therapeutic use [57].

Tumor-Educated Platelets​​​​​

Tumor-educated platelets (TEPs) are critical in local and systemic cancer growth responses. Although they may carry residual mRNA and miRNA from their megakaryocyte ancestors or take up through intercellular contacts in the blood, platelets are typically anucleate. Platelet education is the transfer and sequestration of macromolecules from cancer cells to platelets [58,59]. To induce specific splicing events of pre-messenger RNAs (pre-mRNAs) in circulating platelets, external stimuli in the cancer microenvironment, such as stromal and immune cell signals, can activate platelet surface receptors [60,61]. The diagnostic potential of TEP was initially investigated using mRNA sequencing in individuals with different malignancies [62]. Subsequently, it was discovered that TEPs could reliably identify between benign conditions and early-stage OC [63].

Exosomes

Exosomes’ potential for diagnosis and prognosis has attracted more attention lately. Exosomes are extracellular vesicles with diameter ranging between 30 and 100 nm size, which are extremely stable and resilient to harsh environments and are released by both healthy and malignant cells. They can also be found in various body fluids, including cerebrospinal fluid, ascites, plasma, saliva, and urine [64]. Exosomes can participate in close- and far-reaching signaling by joining the target cell membrane or adhering to the surface receptors. Exosomes can increase carcinogenesis in cancer [65], aid tumor cells in evading the immune system, and result in treatment resistance and therefore can create distant tumor microenvironments, increasing the likelihood of metastasis [66,67]. Exosomes have also been successfully employed as a medicine to eradicate cancer cells [68]. Exosomes are also possible cancer diagnostic indicators since they include proteins, lipids, DNA, and RNA, especially for tumors. MiRNAs can be transported in large amounts by exosomes. Numerous investigations have found variations in exosome miRNA profiles between emergency obstetrics care (EOC) patients and healthy controls. Exosomes from patients with EOC also include more significant levels of melanoma-associated antigen 3 (MAGE3), melanoma-associated antigen 6 (MAGE6), and transforming growth factor-β1 (TGF-β1). Furthermore, claudin 4, connected to cancer antigen 125 (CA125) levels and tumor stage, is more abundant in EOC exosomes. To determine the clinical value of this strategy, in-depth clinical trials are necessary [69].

Clinical applications of liquid biopsy in OC

Emerging research supports the effectiveness of the noninvasive liquid biopsy technique for the detection and long-term surveillance. To provide an earlier diagnosis, a more accurate prognosis, a list of potential molecular targets for therapy, and the ability to spot resistance to treatment, various tumor elements can be examined within acquired plasma samples [66]. It is essential to find OC early to reduce mortality and morbidity. Recurrence-free survival (RFS) is most strongly influenced by stage I or II diseases having much longer RFS than more advanced stages. Therefore, biomarkers that allow the detection of OC in stages II may increase patient outcomes and prolong survival. The prognosis is much worse for two-thirds of EOC cases diagnosed at advanced stages. Interestingly, some researchers have hypothesized that promoter methylation, which leads to epigenetic silencing of tumor suppressor genes, may have diagnostic relevance as an early event in the pathogenesis of OC. Despite the possibility of using aberrant gene promoter methylation to identify cancers, the quantity of collected ctDNA that can be analyzed limits the utility of this technique. Lower ctDNA quantities are associated with earlier stages of EOC, which are often asymptomatic [70]. Exosome analysis and miRNA expression profiling have been shown in some studies to have diagnostic sensitivity and specificity in early-stage disease [71].

Identifying recurrence and establishing prognosis in OC

The clinical use of liquid biopsy is being investigated to determine microscopic residual disease after initial debulking surgery as a prognostic indicator, to predict survival outcomes, and for earlier identification of disease recurrence. Clinically, liquid biopsy can help identify people more likely to relapse so that alternative management strategies can be considered, and they may be eligible for clinical trials. CtDNA provides the most robust data on the predictive value of liquid biopsy. Quantitative ctDNA research reveals a clear correlation between ctDNA concentrations and advanced stages of EOC, which can be used to forecast treatment outcomes [72]. Quantitative polymerase chain reaction (qPCR) and targeted sequencing were used to measure the amounts of ctDNA in 22 EOC patients immediately after surgery. According to the findings of this study, progression-free survival (PFS) and overall survival (OS) were considerably improved when no detectable quantities of ctDNA were detected six months after surgery. All authors came to the same conclusion: Liquid biopsy-based ctDNA analysis has the potential to be used as a clinical biomarker that can predict outcomes, although the small number of samples restricted the majority of studies. The use of ctDNA to identify recurring diseases is also supported by the data. Following first-line therapy, up to 85% of patients with COE may experience recurrence. Patients' chances of survival are limited by EOC recurrence, often described as incurable. Traditional recurrence markers include CA-125 and imaging modalities, including computed tomography (CT) and positron emission tomography (PET) scans [73]. Contrary to conventional imaging methods and CA-125, however, current research has indicated that ctDNA measurement may improve the detection of recurrence [72].

Predicting and Monitoring Treatment Response

After primary debulking surgery and adjuvant chemotherapy, most patients with OC experience complete remission. Nevertheless, the majority of patients experience recurrence due to chemoresistance. According to one theory, the primary factor that causes drug resistance and failure of treatment in OC is intratumor heterogeneity [74]. Intratumor heterogeneity is the term used to describe genomic alterations within a lesion due to cancer cells evolving throughout the multistep carcinogenesis process. It is essential to identify resistance developed in tumor cell clones with time and choose the best-targeted therapy by molecularly analyzing all OC subclones. The capacity of ctDNA to represent tumor heterogeneity helps predict resistance to platinum-based chemotherapy and resistance to PARPi (poly-ADP ribose polymerase inhibitor). A homologous recombination repair defect (HRR) affects roughly half of HGSOC patients, which reduces their ability to repair double-stranded DNA breaks and increases their susceptibility to the DNA damage-inducing alkylating effects of platinum-based chemotherapy. Poly (ADP-ribose) polymerase (PARP)-dependent single-stranded DNA repair processes in BRCA mutant cells make them vulnerable to the synthetic lethality of PARPi. Although platinum-based chemotherapy and PARPi, most OCs with pathogenic BRCA1/2 mutations will eventually develop therapeutic resistance [75].

There are two sorts of mechanisms that cause treatment resistance. One of these is a small sub-clonal cancer call that, after beginning treatment, develops into the primary clone that does not react to targeted therapy and does not carry the BRCA1/2 mutation. The second method effectively restores functional protein synthesis by acquiring reversion mutations close to the original loss-of-function variant [76]. Serial cfDNA samples can be used to longitudinally track the BRCA mutation progression in HGSOC patients receiving PARPi therapy. Previous research has shown that cfDNA from individuals with HGSOC can identify germline and somatic BRCA reversion mutations. To minimize the likelihood of resistance, regular monitoring using liquid biopsy may enable timely diagnosis of resistance and picking more personalized combination therapies (e.g., alternative chemotherapies, targeted therapies, or immunotherapy) targeting various oncogenic drivers [77].

Discussion

Balla et al. (2022) [78] focused on the joint application of CTC and cfDNA methods for molecular profiling, and early diagnosis highlights how liquid biopsies are becoming more and more important in clinical practice. By examining the many uses of liquid biopsies in OC, including early diagnosis, prognostication, molecular targeting, and treatment resistance detection, Zhu et al. (2022) [79] added to the conversation. Their conclusions are given an additional degree of practical consideration by acknowledging the difficulties in ordinary implementation. Wang et al. (2023) [80] explored the general application of liquid biopsy in various cancers and provided a context for the potential effects of OC. Although it is not specifically focused on OC, its view of liquid biopsy as a convenient and effective way of detecting and monitoring cancer contributes to a broader understanding of this diagnostic approach.

Paracchini et al. (2021) [81] limited their focus to monitoring high-grade serous epithelial ovarian cancer (HGS-EOC) with liquid biopsy. Their work highlights the promise of liquid biopsy, providing real-time information on the evolution of the disease and the effectiveness of anti-tumor therapy. Yang et al. (2022) [82] discussed the clinical application of liquid biopsy in OC, emphasizing the role of circulation tumor cells (CTCs) in the development of a comprehensive genomic, transcriptional, and proteomic profile. Their findings suggest that CTCs can inform early diagnosis, predict prognosis, and guide treatment decisions for OC.

Giannopoulou et al. (2018) [83] investigated the potential of liquid biopsy in OC, focusing on miRNA and exosome circulatory as biomarkers. Their research suggests that liquid biopsy can provide accurate medicines with real-time information, and circulatory RNAs and exosomes can be promising biomarkers for diagnosis, prognosis, and treatment. Bhardwaj et al. (2020) [84] discussed the potential application of liquid biopsy in the diagnosis, prognosis, and treatment response of OC. Their findings highlighted the potential of CTCs and tumor DNA as promising biomarkers in the liquid biopsies.

Asante et al. (2020) [85] accepted the promise of liquid biopsies in OC to diagnose, predict, and monitor patients. They emphasized the comprehensive molecular profile and treatment response monitoring with CTCs, further improving the understanding of the development of liquid biopsy applications. Žilovič et al. (2021) [86] discussed the use of liquid biopsy as a non-invasive method for early detection of breast cancer. Although the focus is not explicitly on emerging applications, it brings valuable perspectives, considering the limitations of current diagnostic tools and the promise of liquid biopsy for early detection. Openshaw et al. (2020) [87] presented the latest advances in liquid biopsy for the detection of biomarkers in ovarian and endometrial cancer. Their findings indicate that liquid biopsy may improve cancer detection and monitoring and that extracellular veins reveal treatment markers and targets.

Future directions

There is growing data suggesting that the use of liquid biopsy may improve OC therapy to increase survival. In the previous 10 years, improvements in molecular analysis technology have expanded the therapeutic uses of liquid biopsy for the diagnosis, prognosis, and therapy response [49]. Future studies should use a standardized technique to determine the technical reliability and repeatability of suggested biomarkers within and between laboratories.

## Conclusions

Liquid biopsy is a noninvasive technique, enabling serial samples and longitudinal monitoring to detect treatment resistance as cancer changes over time and direct the choice of individualized therapy. Early-stage OC has low sensitivity and specificity for cfDNA analyses, and only genetic alterations found in cfDNA or ctDNA should be used to inform clinical decision-making in concert with biomarkers and imaging modalities. This technique can be incorporated into clinical studies of OC to determine whether its use will enhance the effectiveness of care and the survival of OC. Before therapeutic application, more research is required to pinpoint the particular release processes, the tissue of origin, and the biological significance of most liquid biopsy components.
